# Exploration and validation of metastasis-associated genes for skin cutaneous melanoma

**DOI:** 10.1038/s41598-022-17468-6

**Published:** 2022-07-29

**Authors:** Hong Luan, Linge Jian, Ye He, Tuo Zhang, Liping Zhou

**Affiliations:** 1 Department of Laboratory Medicine, The First Affiliated Hospital of China Medical University, Shenyang, Liaoning 110001 People’s Republic of China; 2 West China Medical School, Sichuan University, Chengdu, Sichuan 610041 People’s Republic of China; 3 Department of Post Graduation Training, The First Affiliated Hospital of China Medical University, Shenyang, Liaoning 110001 People’s Republic of China

**Keywords:** Cancer, Drug discovery, Immunology, Molecular biology, Molecular medicine

## Abstract

Skin cutaneous melanoma is a malignant and highly metastatic skin tumor, and its morbidity and mortality are still rising worldwide. However, the molecular mechanisms that promote melanoma metastasis are unclear. Two datasets (GSE15605 and GSE46517) were retrieved to identify the differentially expressed genes (DEGs), including 23 normal skin tissues (N), 77 primary melanoma tissues (T) and 85 metastatic melanoma tissues (M). Gene ontology and Kyoto Encyclopedia of Genes and Genomes enrichment analysis were performed to explore the functions of the DEGs. We constructed protein–protein interaction network using the STRING database and Cytoscape software. Using the cytoHubba plugin of Cytoscape, we identified the most significant hub genes by five analytical methods (Degree, Bottleneck, MCC, MNC, and EPC). Hub gene expression was validated using the UALCAN website. Clinical relevance was investigated using The Cancer Genome Atlas resources. Finally, we explored the association between metastasis-associated genes and immune infiltrates through the Tumor Immune Estimation Resource (TIMER) database and performed drug–gene interaction analysis using the Drug-Gene Interaction database. A total of 294 specific genes were related to melanoma metastasis and were mainly involved in the positive regulation of locomotion, mitotic cell cycle process, and epithelial cell differentiation. Four hub genes (CDK1, FOXM1, KIF11, and RFC4) were identified from the cytoHubba plugin of Cytoscape. CDK1 was significantly upregulated in metastatic melanoma compared with primary melanoma, and high CDK1 expression was positively correlated with worse overall survival. Immune infiltration analysis revealed that CDK1 expression negatively correlated with macrophage infiltration (Rho = − 0.164, *P* = 2.02e−03) and positively correlated with neutrophil cells (Rho = 0.269, *P* = 2.72e−07) in SKCM metastasis. In addition, we identified that CDK1 had a close interaction with 10 antitumor drugs. CDK1 was identified as a hub gene involved in the progression of melanoma metastasis and may be regarded as a therapeutic target for melanoma patients to improve prognosis and prevent metastasis in the future.

## Introduction

Skin cutaneous melanoma (SKCM) is an invasive and highly metastatic skin tumor. The 5-year relative survival rate for localized melanoma is 99%, but it is only 25% once tumor metastasis occurs^1^. The American Cancer Society estimated that there were approximately 100,350 newly diagnosed melanoma patients in 2020 in the United States, and the melanoma incidence rate has significantly increased in the past decade worldwide^1,2^. Melanoma has become a serious human health concern, bringing a substantial economic and societal burden. When melanoma is diagnosed at an early stage, surgical resection is the most effective treatment strategy^3^. However, patients suffering from metastatic melanoma have a poor prognosis; therefore, it is necessary to identify new diagnostic biomarkers of melanoma metastasis.

Melanoma is one of the most sensitive tumors to immune regulation^4^. Recently, immunotherapy has proven successful to reduce cancer mortality in advanced stage and metastatic melanoma^5,6^. A recent study showed that the 5-year overall survival rate in Japanese patients with unresectable or metastatic melanoma treated with nivolumab was 26.1%^7^. Due to high mutation rate and tumor heterogeneity^4,8^, primary and secondary drug-resistance still occurs in 60% of metastatic melanoma patients^9^ and the response rates of immune therapy remain low^10^. To explore the exact molecular mechanisms regulating the melanoma metastatic cascade and discovery novel immunotherapeutic targets for melanoma are crucial.

In this study, we compared the mRNA expression between groups of normal skin (N), primary melanoma (T) and melanoma metastasis (M) samples in the GSE15605 and GSE46517 dataset from the Gene Expression Omnibus (GEO) database. The differentially expressed genes (DEGs) were subjected to gene ontology (GO) categories, Kyoto Encyclopedia of Genes and Genomes (KEGG) pathway and protein-protein interaction (PPI) network analyses to determine their functions. We further validated the expression levels and verified the prognostic value of the identified genes using clinical specimens. Finally, immune infiltration and target drugs prediction were further performed to elucidate the potential role of hub genes. Our data provide novel information and help further understand the metastasis cascade of melanoma.

## Materials and methods

### Data collection and processing

Two gene expression datasets of SKCM, namely GSE15605^11^ and GSE46517^12^, were filtered and downloaded from the GEO database (http://www.ncbi.nlm.nih.gov/geo). Among them, GSE15605 consisted of 16 normal skin tissues (N), 46 primary melanoma tissues (T) and 12 metastatic melanoma tissues (M), and GSE46517 contained 7 normal skin tissues, 31 primary tissues and 73 metastatic tissues. The mRNA expression profiles of GSE15605 and GSE46517 were detected using the GPL570 platform (Affymetrix Human Genome U133 Plus 2.0 Array). GEO2R is an online interactive web tool (http://www.ncbi.nlm.nih.gov/geo/geo2r) using the ‘limma’ package of R to screen for differentially expressed mRNAs in T versus N and M versus N. In order to limit the false positive rate, the Benjamini and Hochberg false discovery rate method was selected to adjust the *P* value. adj. *P* < 0.05 and |log2FC|> 1 were set as the cutoff criteria for identifying DEGs.

### GO and KEGG pathway analysis

GO analysis and KEGG enrichment were performed on the DEGs, and the results were visualized with the R packages of ‘GOplot’, ‘DOSE’, ‘enrichplot’ and ‘clusterProfiler’. *P* < 0.05 was used as the cutoff criterion.

### PPI network construction and hub gene selection and analyses

The Search Tool for the Retrieval of Interacting Genes^13^ (STRING, http://string-db.org) (version 11.5) was utilized to supply the PPI network data of 294 DEGs. Those with an interaction score ≥ 0.7 were considered as the cutoff criterion^13^. Then, the string interaction networks were imported into Cytoscape software^14^ (http://www.cytoscape.org) (version 3.7.2) for visualization. Specifically, we applied the Cytoscape plugin CytoHubba^15^ to identify key genes via five analytic methods: maximal clique centrality (MCC), maximum neighborhood component (MNC), Degree, edge percolated component (EPC) and Bottleneck.

### Validation of hub gene expression

The UALCAN website (http://ualcan.path.uab.edu/analysis.html), as a database for analyzing TCGA data and clinical patient information, was used to investigate the mRNA expression of hub genes^16^. A total of 473 samples, including 1 normal tissue, 104 primary melanomas and 368 metastatic melanomas, were contained. *P* < 0.05 was statistically significant. Protein expression data of hub genes in primary melanoma tissues and metastasis tissues were evaluated using the Human Protein Atlas tool (HPA, https://www.proteinatlas.org/)^17^.

### Survival analysis of hub genes by TCGA

Then, based on the median expression level for each gene, patients with melanoma were classified into high expression group and low expression group, and we performed survival analysis and plotted Kaplan–Meier curve using Gene Expression Profiling Interactive Analysis (GEPIA, http://gepia.cancer-pku.cn/index.html) database. The overall survival (OS) curves of hub genes were also drawn by the UALCAN website, and *P* < 0.05 was considered as significant value.

Meantime, univariate- and multivariate Cox regression analysis were performed to screen the prognosis-related gene significantly associated with OS in the TCGA SKCM and GSE46517 dataset with *P* < 0.05 as the criterion. The TCGA SKCM gene expression data and clinic data were downloaded from the UCSC Xena database (https://xena.ucsc.edu/). A total of 454 melanoma samples were matched with corresponding patients with complete survival information in the TCGA dataset and 48 specimens had complete survival data in GSE46517. Hazard ratios and corresponding confident intervals were calculated through R package ‘survival’.

### Immune infiltration analysis

The relationship between metastasis-associated gene expression and the abundance of immune cell infiltration in SKCM metastasis was evaluated in the Tumor Immune Estimation Resource (TIMER)^18^. TIMER included six immune cells: B cells, CD4+ T cells, CD8+ T cells, neutrophils, macrophages, and dendritic cells. *P* < 0.05 was identified to be significant.

### Drug-gene interaction networks analysis

The Drug-Gene Interaction database (DGIdb, version 4.2.0-sha1 afd9f30b, http://www.dgidb.org/)^19^ was established to predict the interaction networks between hub gene and related therapeutic drugs. In our study, we regarded the metastasis-associated genes as promising drug targets and searched for the potentially druggable category using DGIdb, with the parameters set as follows: preset filters: antineoplastic; all the default. After the prediction of drug–gene pairs related to the metastasis-associated genes, the network was then constructed using Cytoscape.

## Results

### Screening for DEGs

**Figure 1:**
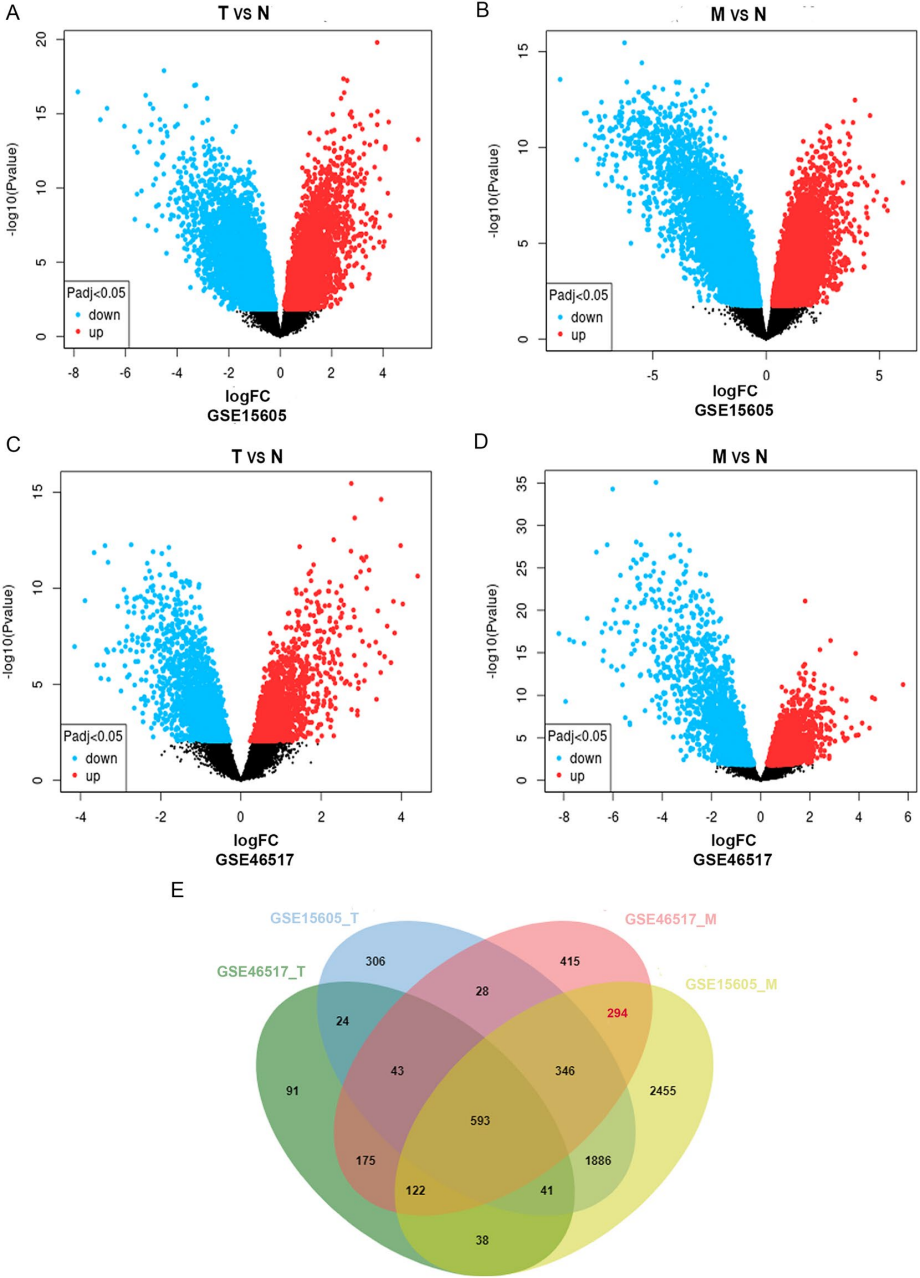
Identification of specific genes associated with metastasis in melanoma. (**A**,**B**) Differentially expressed genes (DEGs) were screened by volcano plot comparing 16 normal skin (N), 46 primary melanoma (T) and 12 metastatic melanoma tissues (M) from GSE15605. (**C**,**D**) DEGs were screened by volcano plot when comparing 7 N samples, 31 T samples and 73 M samples in GSE46517. (**E**) Venn diagram for overlapping DEGs in 4 microarray datasets. |log2 FC|> 1 and adj. *P* < 0.05 were set as the cutoff criterion.

To identify the metastasis-associated gene signature in melanoma, the mRNA expression levels was compared in T versus N and M versus N in GSE15605 and GSE46517 dataset, respectively. |log2FC|> 1 and *P* < 0.05 were defined as statistically meaningful cutoff points. When analyzing the gene expression between T versus N, a total of 3267 DEGs, including 1444 upregulated and 1823 downregulated transcription factors, were filtered in GSE15605, and a total of 1127 DEGs, consisting of 527 upregulated and 600 downregulated transcription factors, were identified in GSE46517 (Fig. 1a,c). A total of 5775 DEGs, containing 3017 upregulated and 2758 downregulated in M versus N in GSE15605. The volcano plot of DEGs distribution presented 2016 genes, including 952 upregulated and 1064 downregulated DEGs, when comparing M with N in GSE46517, (Fig. 1b,d). As shown in the Venn diagram (Fig. 1e), a total of 1355 overlapped genes were obtained between the two metastasis-associated datasets, which might be involved in the pathophysiological process of melanoma metastasis, but except for 1061 genes related to tumor development, 294 genes were specific for melanoma metastasis.

### Functional enrichment analysis for DEGs

**Figure 2:**
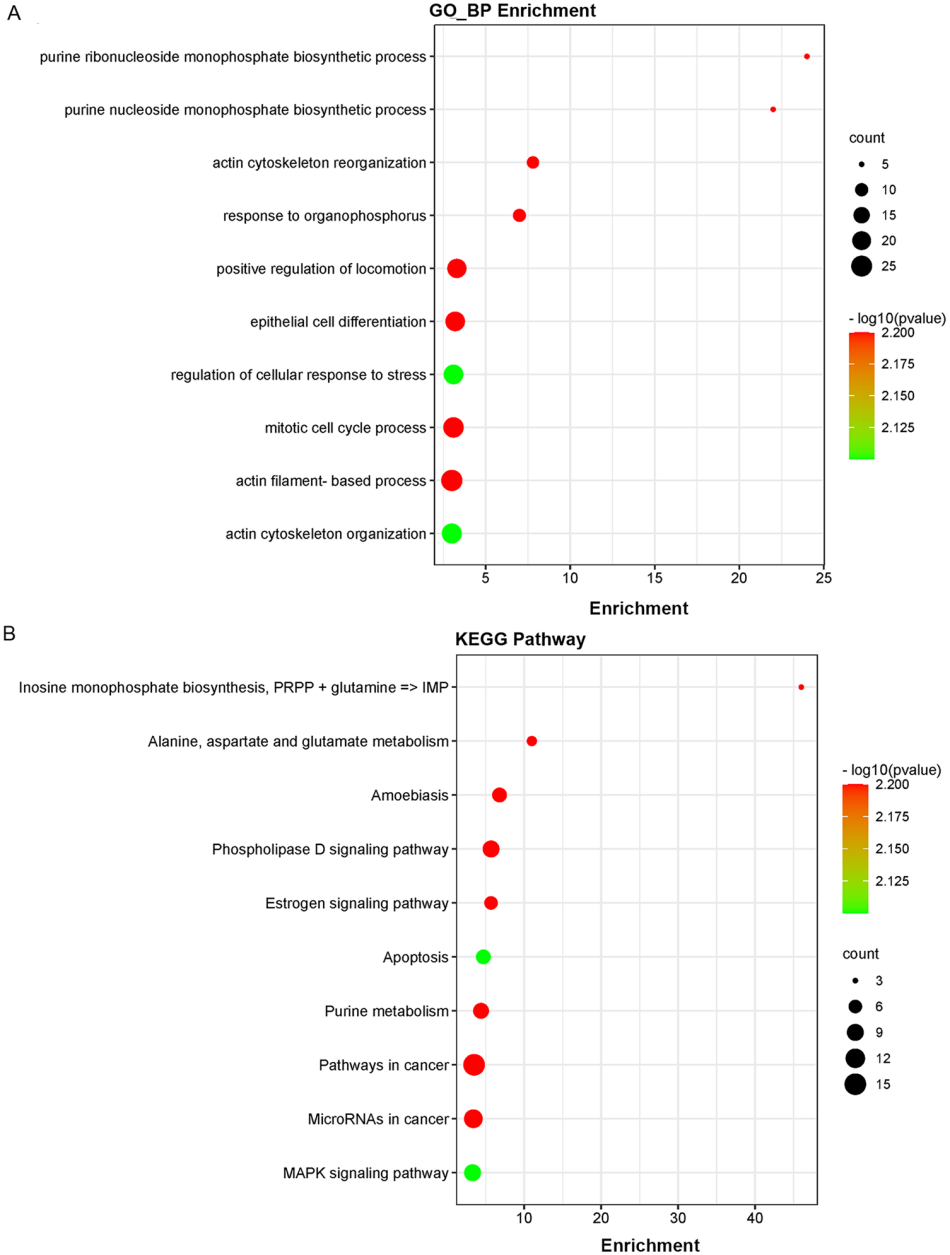
GO and KEGG pathway enrichment analysis of DEGs. (**A**) Top ten enriched biological processes for the DEGs. (**B**) Top ten enriched KEGG pathways for the DEGs.

All 294 screened DEGs were subjected to GO and KEGG pathway analysis by the R software. In GO biological process analysis, DEGs were mainly concentrated on positive regulation of locomotion, mitotic cell cycle process, epithelial cell differentiation, and actin filament-based process. In KEGG pathway analysis, DEGs were dominant enriched in pathways in cancer, microRNAs in cancer, phospholipase D signaling pathway, and purine metabolism (Fig. 2).

### PPI network analysis and hub Gene screening

**Figure 3:**
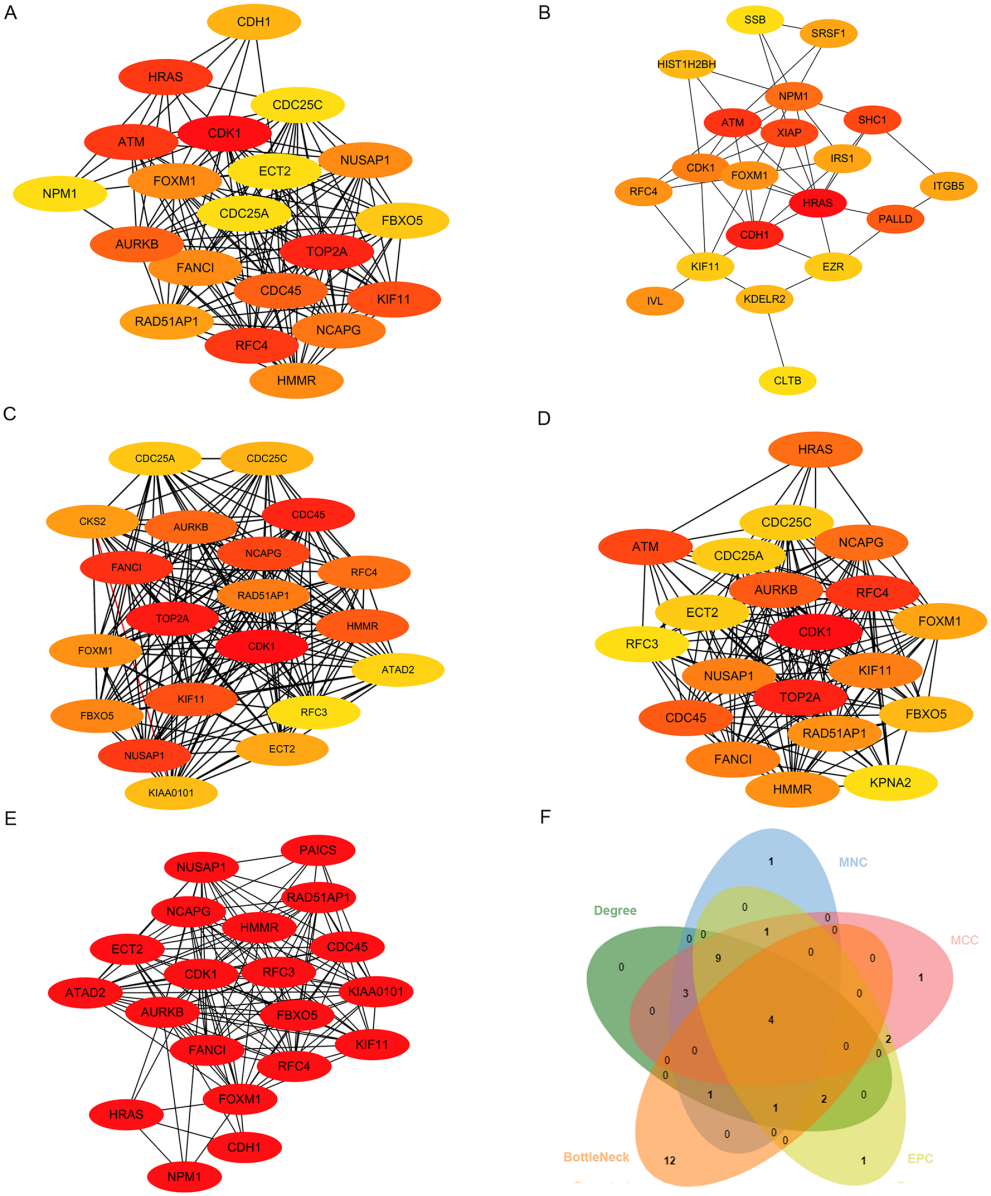
Identification of hub genes. (**A**–**E**) The hub genes were identified using five topological analysis methods (**A**) Degree, (**B**) Bottleneck, (**C**) MCC, (**D**) MNC, and (**E**) EPC with cytoHubba (**F**) A Venn diagram showed that four hub genes were identified.

The STRING database was used to draw the PPI network diagram of the DEGs. DEGs with an interaction score ≥ 0.7 were eligible for constructing the relational network. We ranked the top 20 genes of the whole network based on the Cytoscape plugin CytoHubba models: Degree, MCC, MNC, EPC and Bottleneck (Fig. 3a–e). Venn analysis was performed to obtain the intersection of these genes. Notably, the top 20 genes from topological analysis algorithms included four hub genes: CDK1, FOXM1, KIF11, and RFC4, which may involve in the development of metastatic melanoma (Fig. 3f).

### Validation of hub genes expression

**Figure 4:**
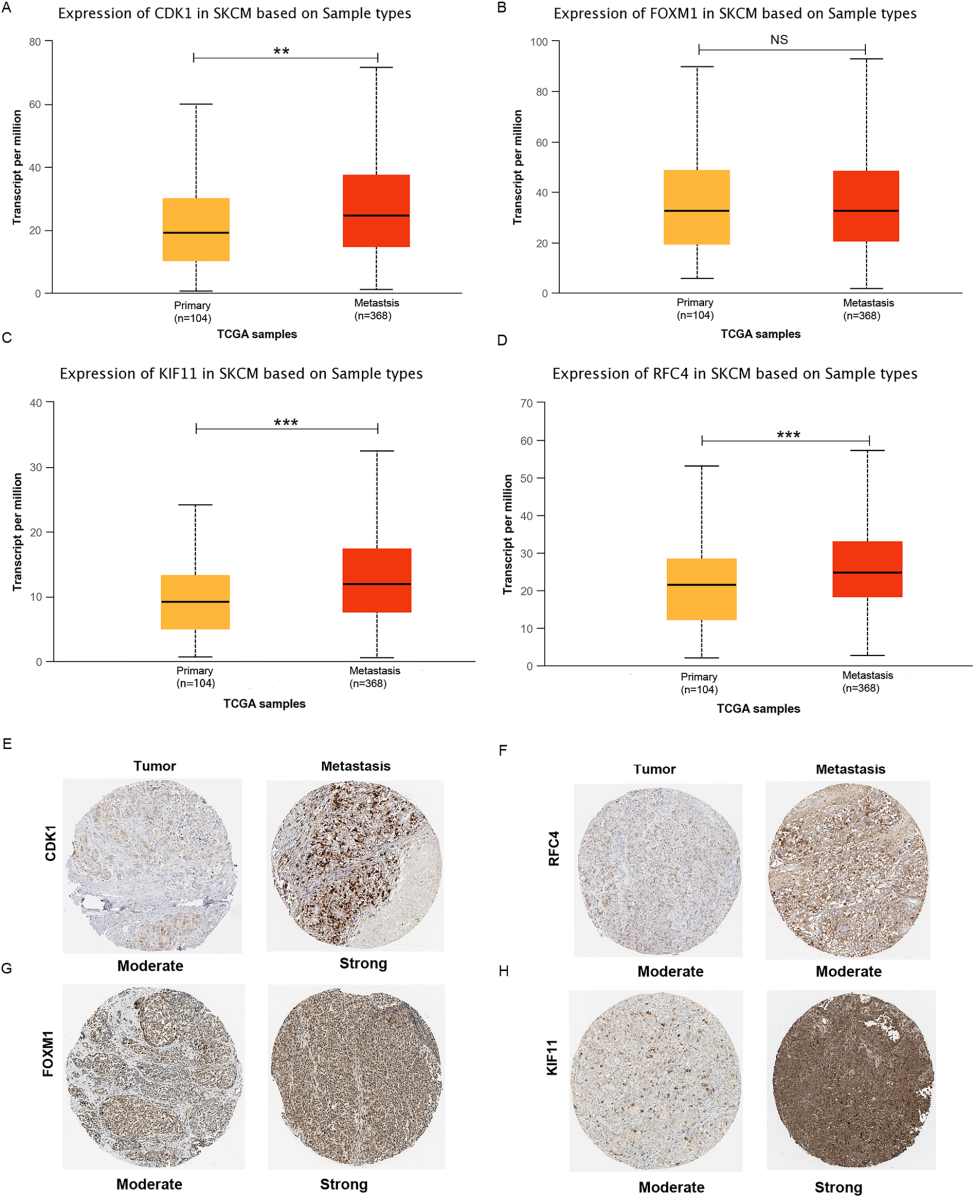
Validation of hub genes expression in primary and metastatic melanoma samples. (**A–D**) Box plot of hub genes mRNA expression level were obtained by UALCAN platform. *P < 0.05;**P < 0.01; ***P < 0.001. Abbreviation: NS, no significance. (**E**–**H**) Immunohistochemical staining of CDK1, RFC4, FOXM1 and KIF11 in primary melanoma and metastatic melanoma tissues using the HPA database in SKCM.

To further verify previously defined hub genes, we obtained expression data of 472 TCGA-SKCM samples, including 104 primary melanomas and 368 metastatic tissue samples from the UALCAN database. The results showed that three hub genes (CDK1, KIF11 and RFC4) were significantly upregulated in metastatic melanoma tissues compared in primary melanoma tissues (Fig. 4a–d). Significant differences are displayed as follows: *P < 0.05; **P < 0.01; ***P < 0.001. FOXM1 expression was not significantly different between primary melanoma tissues and metastatic melanoma tissues (*P* > 0.05). To explore the protein expression level of four hub genes in SKCM, we conducted immunohistochemistry analysis of protein expression using HPA database. As shown in Fig. 4e–h, we found that except for the moderate staining of RFC4 in both primary and metastasis tissues of the melanoma, the other three hub genes showed moderate expression in primary tumor tissues, but showed strong expression in metastatic melanoma tissues.

### Prognosis value of hub genes in SKCM using GEPIA database and UALCAN database

**Figure 5:**
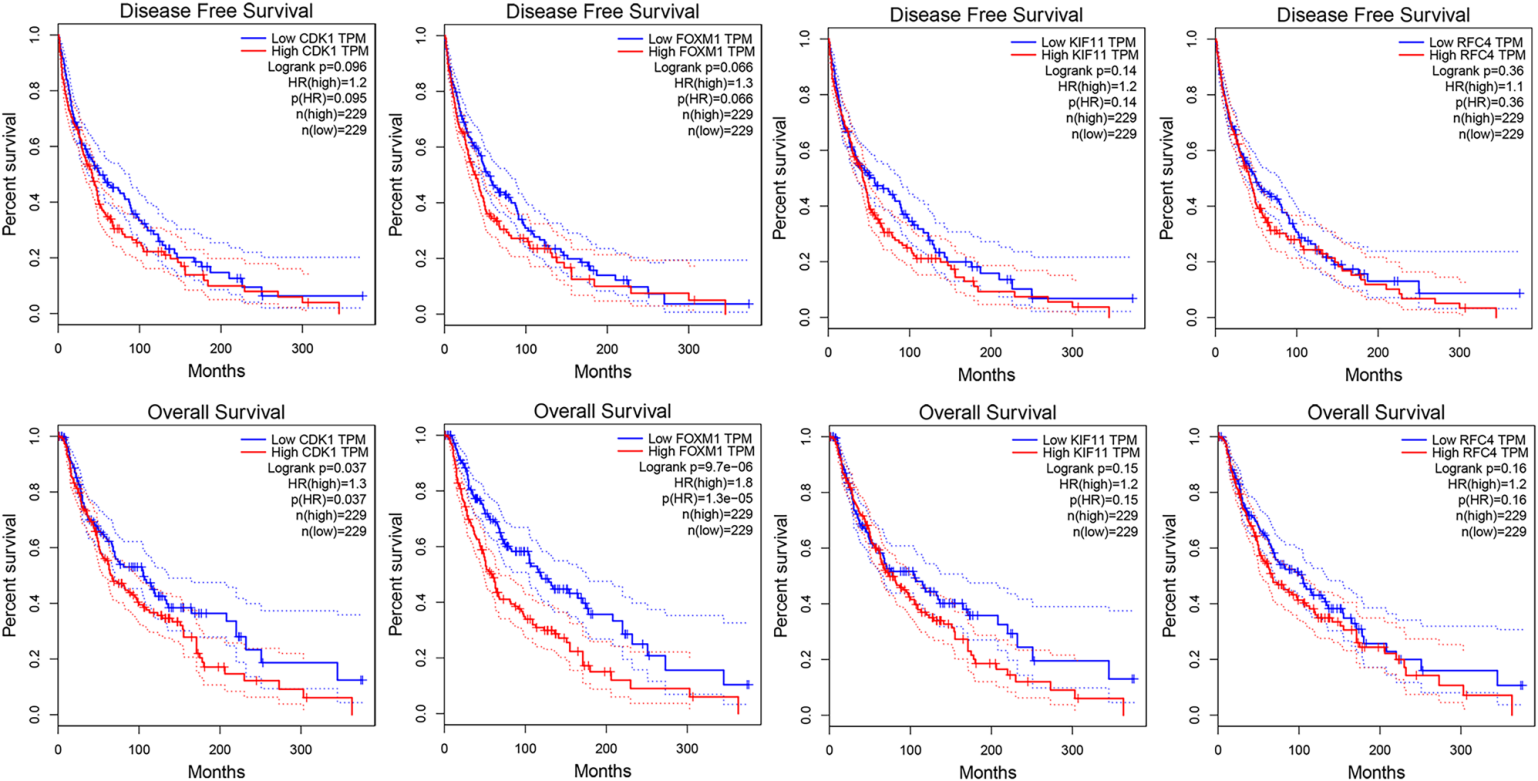
Association of hub gene expression levels and prognosis of SKCM based on the TCGA SKCM datasets by GEPIA. *P* < 0.05 was considered statistically significant.

To assess the effect of hub gene expression on the prognosis of SKCM, we performed survival analysis to identify the association of hub genes with overall survival (OS) and disease-free survival (DFS) in the GEPIA database. As shown in Fig. 5, The up-regulated expression of two hub genes (CDK1: OS *P* = 0.037; FOXM1: OS *P* = 9.7e−06) were positively correlated with poor prognosis. The expression of KIF11 and RFC4 had no correlation with OS and DFS. To confirm the results, the relationships between four hub genes and OS were investigated using the UALCAN database. The results suggested that melanoma patients with high expression of CDK and FOXM1 had shorter OS (CDK1: OS *P* = 0.047; FOXM1: OS *P* = 0.00043) (Fig. S1).

To further verify the prognostic value of hub genes in the patients with melanoma, Cox regression analysis was performed by univariate and multivariate on the TCGA and GSE46517 dataset. The results indicated that FOXM1 was significantly associated with OS and was a risk factor with HR > 1 in the TCGA-SKCM cohort (univariate Cox: HR = 1.31, 95% CI 1.15–1.5, *P* < 0.001; and multivariate Cox: HR = 1.53, 95% CI 1.31–1.80, *P* < 0.001). In multivariate analysis, CDK1 showed that HR > 1 and the *P* value was close to 0.05 (HR = 1.23, 95% CI 0.98–1.55, *P* = 0.053), suggesting that high CDK1 was correlated with poor prognosis of melanoma. Other two hub genes were not correlated with overall survival of patients with melanoma. Due to the limited sample size, the difference was not significant in GSE46517 (Fig. S2).

### Immune infiltration analysis

**Figure 6:**
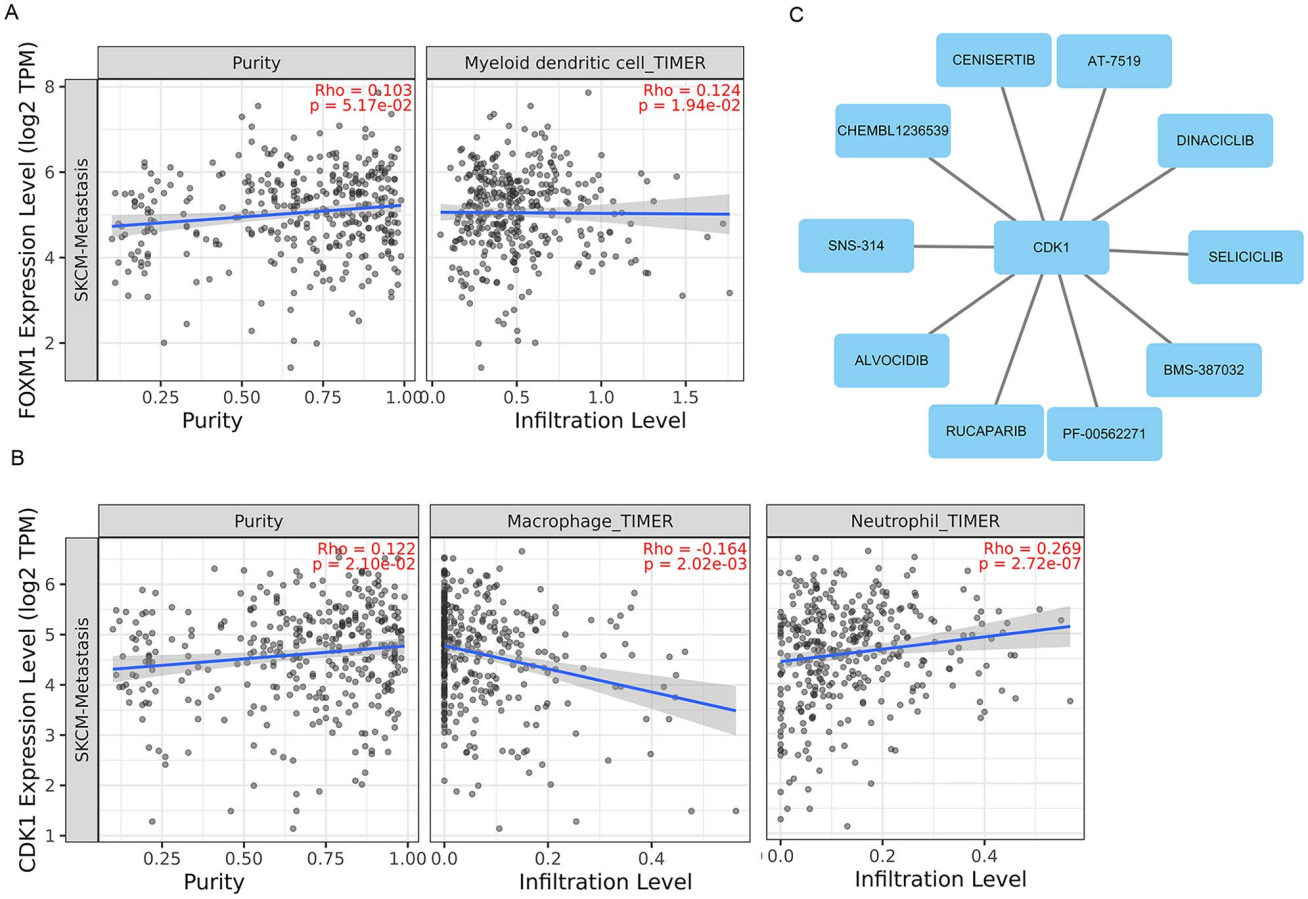
Hub gene analysis. (**A**) Correlation analyses of FOXM1 expression with the infiltration degree of myeloid dendritic cells in SKCM metastasis through TIMER database. (**B**) Correlation analyses of CDK1 expression and immune infiltrates (neutrophils and macrophages) in SKCM metastasis through TIMER database. (**C**) Drug-hub gene interaction network. Blue nodes represented the drug. The line represents the interaction relationship between CDK1 and the drug.

We analyzed the correlations between CDK1 and FOXM1 expression and the immune infiltration levels of six immune cells (CD8+ T cells, CD4+ T cells, B cells, neutrophils, macrophages and myeloid dendritic cells) in the SKCM metastasis microenvironment using TIMER. *P* < 0.05 was considered as significance. We found that FOXM1 expression was positively correlated with the infiltration degree of myeloid dendritic cells (Rho = 0.124, *P* = 1.94e−02) (Fig. 6a, S3). CDK1 expression was negatively correlated with infiltrating levels of macrophage cells (Rho = − 0.164, *P* = 2.02e−03) and positively correlated with the infiltration degree of neutrophil cells (Rho = 0.269, *P* = 2.72e-07) (Fig. 6b). The expression of CDK1 was not associated with the immune infiltration levels of CD8+ T cells, CD4+ T cells, B cells or dendritic cells (Fig. S3).

### Drug–gene interaction networks analysis

DGIdb database was used to predict the potential targeted drugs that interacted with the CDK1 and FOXM1 genes. We identified 10 drugs that interacted with CDK1; however, there was no drug that interacted with FOXM1, as shown in Fig. 6c. These results may reveal the therapeutic targets related to metastatic melanoma.

## Discussion

Tumor metastasis is the most common recurrence mode and cause of death in SKCM, which seriously affects patient survival and prognosis. Thus, it is urgent to research the molecular mechanism of promoting melanoma invasion and explore effective indicators for monitoring this pathophysiological process.

In the current study, we screened 294 genes that were closely related to melanoma metastasis. GO analysis showed that DEGs were mainly enriched in the positive regulation of locomotion, mitotic cell cycle process, epithelial cell differentiation, and actin filament-based process. The above biological processes are mainly related to cell movement and cell proliferation. We speculated that DEGs induced melanoma cell metastasis by promoting cell migration and cell proliferation. KEGG enrichment analysis revealed that DEGs were mainly enriched pathways in cancer, microRNAs in cancer, phospholipase D signaling pathway, and purine metabolism. Phospholipase D is an enzyme that catalyzes the hydrolysis of phosphatidylcholine to phosphatidic acid. Studies have found that the phospholipase D signaling pathway is involved in lung cancer-mediated bone metastasis^20^, and phospholipase D isoform 1 promotes tumor invasion of bladder cancer by regulating MMP-13 expression^21^.The role of the phospholipase D signaling pathway in melanoma metastasis is worth further exploration.

Next, we identified 4 specific metastasis-associated genes by conducting a PPI network and analyzed their prognostic value through the TCGA database. The results suggested that CDK1 and FOXM1 may serve as favorable predictive factors for melanoma patient survival. FOXM1 is involved in mediating myriad biological processes, including cell proliferation, cell cycle progression, cell differentiation, DNA damage repair, tissue homeostasis, angiogenesis and apoptosis, and has been suggested to be a key player in tumorigenesis^22^. Moreover, FOXM1 is a known downstream factor of the Akt signaling cascade^23,24^, and inactivation of the Akt/FOXM1 signaling pathway may suppress the proliferation and metastasis of gastric cancer cells^25^. In our present study, we found that FOXM1 expression was not significantly different between metastatic melanoma tissues and primary melanoma tissues, and FOXM1 seemed not to be a potential metastasis-associated biomarker for melanoma. We therefore further researched the CDK1 gene, which is specifically involved in the metastasis process of melanoma and predicts a bad prognosis.

CDK1, namely, cyclin-dependent kinase 1, is a serine/threonine-like protein kinase and plays critical roles in the regulation of the cell cycle and cell proliferation. Previous studies have reported that CDK1 forms a complex with cyclin A/B that is involved in regulating cell mitosis, G2/M checkpoint maintenance, execution of apoptosis, and genomic stability maintenance^26^. Furthermore, CDK1 has been revealed to be overexpressed in various malignancies. High-CDK1-expression colorectal cancer patients had a poor clinical outcome, and inhibition of CDK1 enhances 5-fluorouracil sensitivity in colorectal cancer^27^. Huang et al*.* reported that CDK1/Sox2 axis was responsible for regulating and maintaining the stemness of lung cancer cells, and inhibition of CDK1 enhanced chemotherapeutic sensitivity in lung cancer^28^. Recently, CDK1-mediated phosphorylation of TFCP2L1 has been proved to be required for stem cell pluripotency and bladder carcinogenesis^29^. CDK1 promoted tumor initiation in human melanoma through interacting with Sox2^30^. The role of CDK1 in melanoma metastasis has not been reported previously.

The tumor microenvironment (TME) is composed of immune cells, mesenchymal cells, endothelial cells, inflammatory mediators and extracellular matrix (ECM) molecules^31,32^. The composition and abundance of immune cells in the tumor microenvironment strongly influence the progression of tumors and the effect of immunotherapy^33–35^. We found that the expression levels of CDK1 were correlated with immune cell infiltration. We speculate that CDK1 is involved in the process of melanoma metastasis by regulating the function of the tumor microenvironment. Therefore, the function and pathway of CDK-mediated immune cell-infiltration need to be further investigated.

Currently, according to the genotype and stage of melanoma, targeted therapy, such as BRAF inhibitors and MEK inhibitors, has been used as first-line treatment or adjuvant therapy for patients^36^. In 2011, the FDA approved the first targeted therapeutic drug for advanced BRAF-mutant melanoma, vemurafenib^37^. In this study, ten drugs were identified and they may serve as potential therapeutic targets. Rucaparib, as a PAPP inhibitor, has been approved by the FDA for the clinical treatment of ovarian cancer^38^. In a phase II study, temozolomide (150–200 mg/m(2)/day) was safely given withrucaparib, increasing progression-free survival over historical controls in patients with advanced metastatic melanoma^39^. Dinaciclib induces p53 expression while simultaneously downregulating the expression of the antiapoptotic factors Mcl-1 and XIAP in melanoma cell lines^40^. The small-molecule drug AT-7519 diminished MDM4 levels and activated p53 in the A375 melanoma cell line^41^. Other drugs have not been utilized to cure melanoma metastasis in the literature.

The main limitation of our study was only the analysis of bioinformatics, so it was urgent to carry out cytological experiments, animal experiments, and drug trials to verify these hub genes in melanoma metastasis.

## Conclusions

In summary, 294 DEGs between the primary and metastatic skin cutaneous melanoma samples were screened, and four hub genes, CDK1, FOXM1, KIF11, and RFC4, were identified that may be associated with the metastasis of melanoma. UALCAN suggested that CDK1 was significantly upregulated in metastatic melanoma compared with primary melanoma and that high expression of CDK1 was positively correlated with poor prognosis. Moreover, CDK1 plays an important role in the microenvironment of metastatic melanoma by regulating the tumor infiltration of immune cells. Ten candidate small molecule antitumor drugs were selected. Further studies are needed to investigate these metastasis-associated genes as therapeutic targets.

## Supplementary Information

Supplementary Information.

## Data Availability

The data used to support the findings of this study were available from the Gene Expression Omnibus (http://www.ncbi.nlm.nih.gov/geo) and Cancer Genome Atlas database (https://cancergenome.nih.gov).

## Abbreviations

SKCM: Skin cutaneous melanoma
GEO: Gene Expression Omnibus
DEG: Differentially expressed gene analyses
GO: Gene ontology
KEGG: Kyoto Encyclopedia of Genes and Genomes
PPI: Protein–protein interaction
GEPIA: Gene Expression Profiling Interactive Analysis
DGIdb: Drug–Gene Interaction database
TIMER: Tumor Immune Estimation Resource
OS: Overall survival
DFS: Disease-free survival
TME: Tumor microenvironment
