# Supplementary material for: Exploration and validation of metastasis-associated genes for skin cutaneous melanoma
Source: Sci Rep. 2022 Jul 29;12:13002. doi: 10.1038/s41598-022-17468-6 (PMC9338051; doi:10.1038/s41598-022-17468-6)
Supplement: Supplementary file 1 — Supplementary Information. [file 41598_2022_17468_MOESM1_ESM.docx]

**Supplementary Materials**


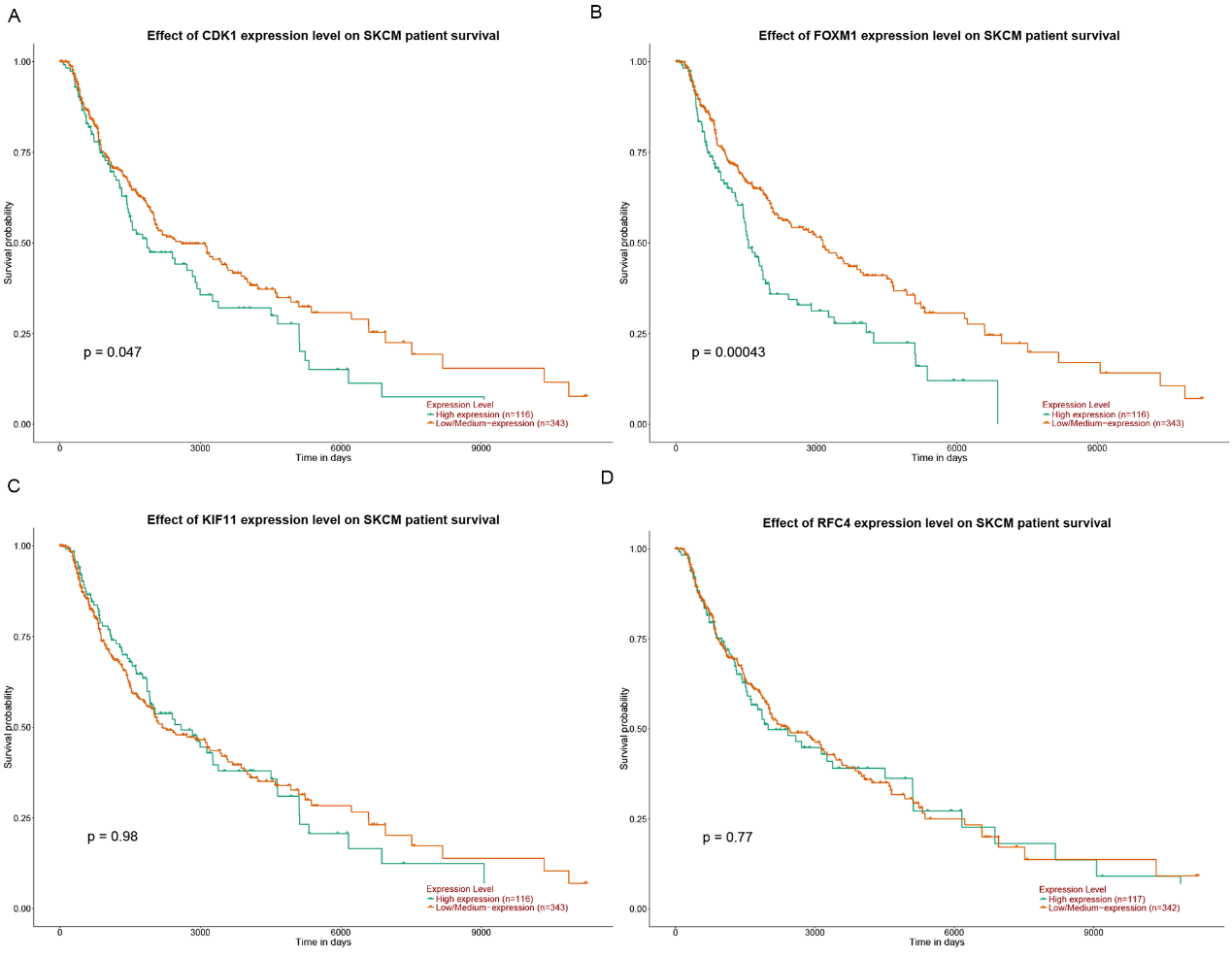


**Figure S1:** Overall survival analysis of hub genes in SKCM was performed by using the UALCAN platform.


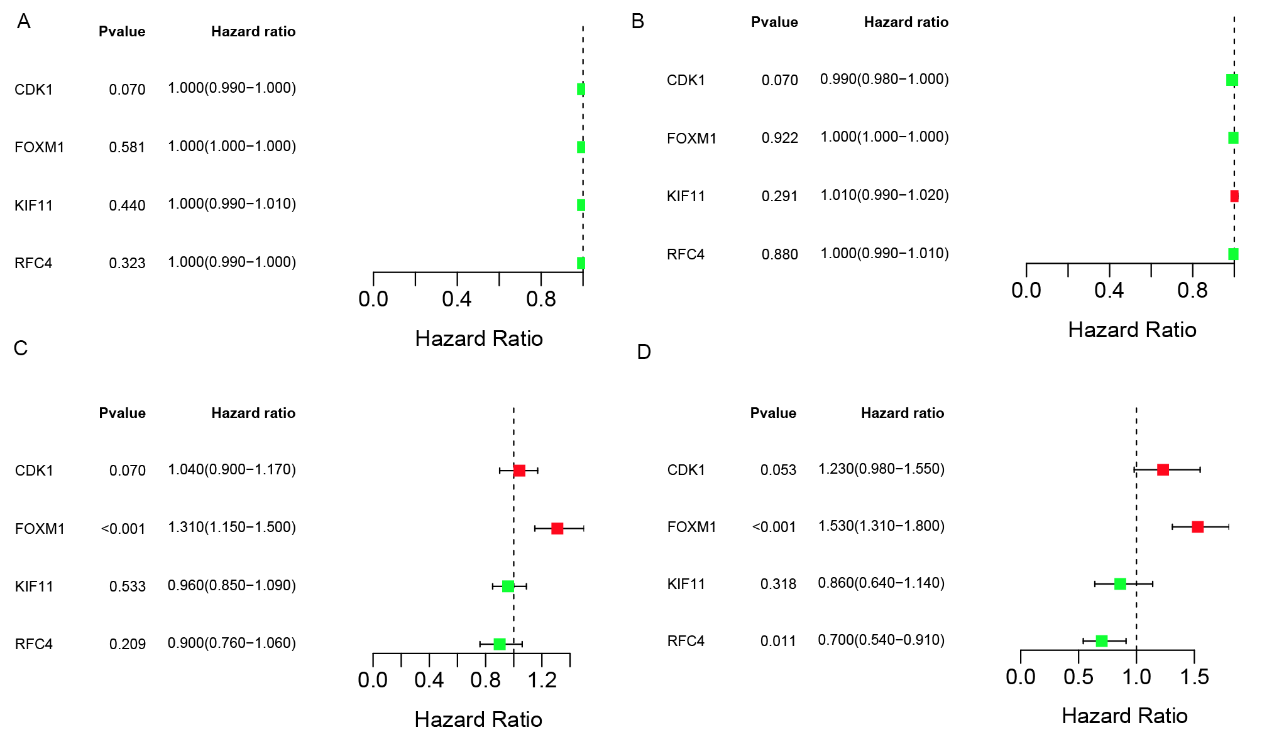


**Figure S2:** Verify the prognostic value of hub genes in the patients with melanoma. Univariate (A)and multivariate (B) Cox regression analysis of four hub genes with OS in GSE46517 from GEO database. Univariate (C)and multivariate (D) Cox regression analysis of four hub genes with OS in the TCGA-SKCM cohort.


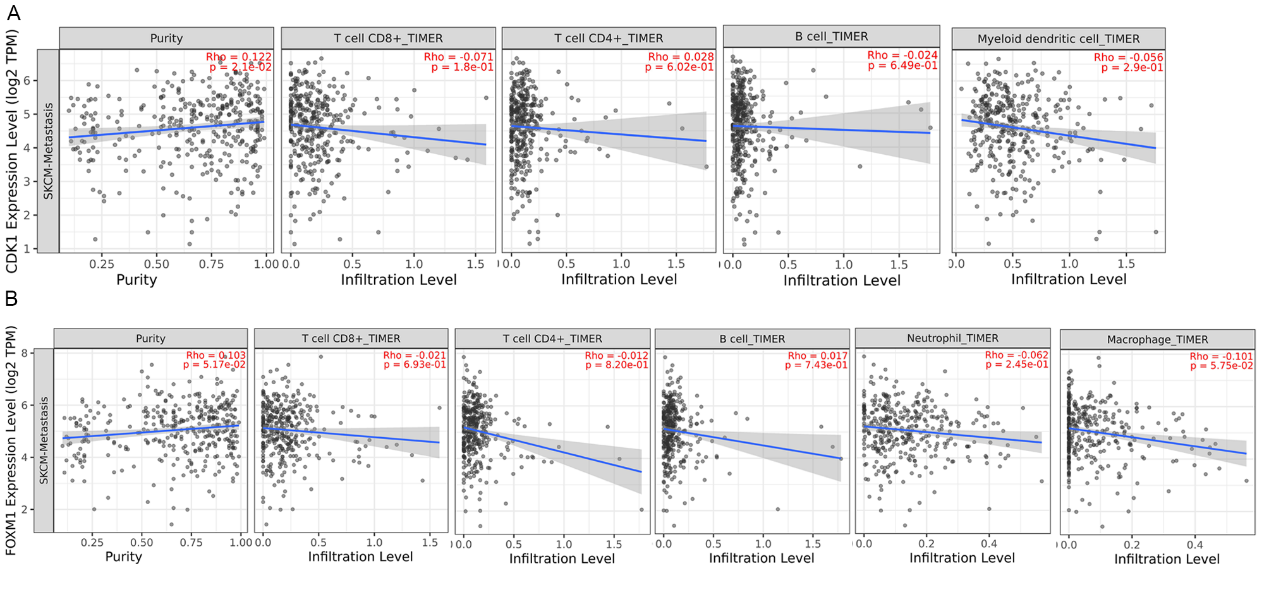


**Figure S3:** Correlation analyses of CDK1 (A) and FOXM1 (B) expression and immune infiltrates (B cells, CD4+ T cells, CD8+ T cells, neutrophils and macrophages) in SKCM metastasis through the TIMER database.
